# A Review of Medicinal Plants with Antiviral Activity Available in Bangladesh and Mechanistic Insight Into Their Bioactive Metabolites on SARS-CoV-2, HIV and HBV

**DOI:** 10.3389/fphar.2021.732891

**Published:** 2021-11-08

**Authors:** Sitesh C. Bachar, Kishor Mazumder, Ritesh Bachar, Asma Aktar, Mamun Al Mahtab

**Affiliations:** ^1^ Department of Pharmacy, Faculty of Pharmacy, University of Dhaka, Dhaka, Bangladesh; ^2^ Department of Pharmacy, Jashore University of Science and Technology, Jashore, Bangladesh; ^3^ School of Optometry and Vision Science, UNSW Medicine, University of New South Wales (UNSW), Sydney, NSW, Australia; ^4^ School of Biomedical Sciences and Graham Centre for Agricultural Innovation, Charles Sturt University, Wagga, NSW, Australia; ^5^ Department of Pharmacy, School of Science and Engineering, University of Information Technology and Sciences, Dhaka, Bangladesh; ^6^ Department of Hepatology, Bangabandhu Sheikh Mujib Medical University, Dhaka, Bangladesh

**Keywords:** antiviral drug discovery, medicinal plants, viral diseases, natural antiviral, SARS-CoV-2

## Abstract

Currently, viral infection is the most serious health issue which causing unexpected higher rate of death globally. Many viruses are not yet curable, such as corona virus-2 (SARS-CoV-2), human immunodeficiency virus (HIV), hepatitis virus, human papilloma virus and so others. Furthermore, the toxicities and ineffective responses to resistant strains of synthetic antiviral drugs have reinforced the search of effective and alternative treatment options, such as plant-derived antiviral drug molecules. Therefore, in the present review, an attempt has been taken to summarize the medicinal plants reported for exhibiting antiviral activities available in Bangladesh along with discussing the mechanistic insights into their bioactive components against three most hazardous viruses, namely SARS-CoV-2, HIV, and HBV. The review covers 46 medicinal plants with antiviral activity from 25 families. Among the reported 79 bioactive compounds having antiviral activities isolated from these plants, about 37 of them have been reported for significant activities against varieties of viruses. Hesperidin, apigenin, luteolin, seselin, 6-gingerol, humulene epoxide, quercetin, kaempferol, curcumin, and epigallocatechin-3-gallate (EGCG) have been reported to inhibit multiple molecular targets of SARS-CoV-2 viral replication in a number of *in silico* investigations. Besides, numerous *in silico*, *in vitro*, and *in vivo* bioassays have been demonstrated that EGCG, anolignan-A, and B, ajoene, curcumin, and oleanolic acid exhibit anti-HIV activity while piperine, ursolic acid, oleanolic acid, (+)-cycloolivil-4′-O-β-d-glucopyranoside, quercetin, EGCG, kaempferol, aloin, apigenin, rosmarinic acid, andrographolide, and hesperidin possess anti-HBV activity. Thus, the antiviral medicinal plants and the isolated bioactive compounds may be considered for further advanced investigations with the aim of the development of effective and affordable antiviral drugs.

## Introduction

Currently, viral infection has come to be the major global challenge to healthcare professionals due to uncontrolled rate of morbidity as well as mortality. A number of life-threatening viruses including human immunodeficiency virus (HIV), hepatitis virus subtype A, B, and C (HAV, HBV, and HCV), herpes simplex virus (HSV), influenza virus, and so others have been affected human health for decades. Along with these pre-existing viruses, corona virus-2 (SARS-CoV-2) has been turning into a global burden from 2019. The corona virus infection, also termed as “the novel coronavirus disease” (COVID-19) is characterized by severe acute respiratory syndrome resulting very high rate of death (Gisondi et al., 2020). Unfortunately, lack of safe as well as effective antiviral drugs against these viruses has worsened the situation.

Over past few decades, advanced scientific research has discovered many synthetic antiviral agents which are effective against many of the viral infectious diseases. Unfortunately, these synthetic drugs have been reported to produce countless adverse effects. In some cases, they may become ineffective on emerging viral resistant strains (Kurokawa et al., 2010). Along with this, the population in developing countries can’t afford these expensive synthetic medicines for treatment of viral diseases. Keeping in view the global burden of viral infections as well as medication cost, there is an urgent need to develop new strategies to search for affordable and effective antiviral drugs.

Ethnopharmacology has contributed immensely to the development of phytotherapeutics and the discovery of new drugs (Heinrich and Gibbons, 2001). In recent time, medicinal plants and their bioactive metabolites have become one of the main focuses of interest to search for effective as well as affordable drugs to cope with the current necessities (Perera and Efferth, 2012). Traditional herbal medicine from indigenous origin has an ancient history of curing numerous chronic and infective diseases. Hence, the quest for novel antiviral agents focuses not only on synthetic combinations but also on the plant-derived metabolites. A variety of plant metabolites can impede viral replication without affecting the host physiology or with limited side effects (Martin and Ernst, 2003; Hussain et al., 2017). Along with direct interferences to viral replication process, these natural products may exhibit potentiality to modulate the immune responses of host against viral infections (Kurokawa et al., 2010). Researchers have reported that numerous medicinal plants with antiviral activities, such as *Andrographis paniculata*, *Lindera chunii*, *Dioscorea bulbifera*, *Wistaria floribunda*, *Xanthoceras sorbifoli, and Aegle marmelos* showed remarkable anti-HIV activity (Kaur et al., 2020). Moreover, a number of natural or plant-derived compounds belonging to different chemical groups have been reported for their potential anti-HBV activities (Chou et al., 2012; Qiu and Chen, 2013; Parvez et al., 2016; Wu, 2016). Some plant products have shown similar or even better efficacy against this virus than that of interferons and/or lamivudine treatment (Chen and Zhu, 2013; Arbab et al., 2017; Zhang et al., 2017). Interestingly, therefore, approximately 80% of the chronic hepatitis B (CHB) patients in China rely on traditional herbal medicines.

There is much to gain and learn about remedial qualities of plants from the pre-existing knowledge of traditional medicines that may be evaluated for various applications as potential antiviral drugs. It is convenient to find plants that can be researched upon; however, what is required is the traditional knowledge that must be translated into pharmaceutical application in formulating novel drugs, finally taking it from the laboratory bench to the bedside. Even though numerous medicinal plants as well as plant derived metabolites have been reported for their antiviral effects, there lacks adequate combined substantial reports of pre-existing researches with mechanistic insights (Martin and Ernst, 2003). In most of the cases, due to lack of any substantial compilation report, the researchers conducted the similar studies as preliminary screening prior to design the advanced stages of discovery of potent drug molecule from plant. This is a complete loss of time, money and efforts. Therefore, an attempt is taken to review the medicinal plants indigenous to and/or cultivated in Bangladesh having antiviral activities along with emphasizing mechanistic insights of their bioactive metabolites on viral replication cycles of the most hazardous viruses, like SARS-CoV-2, HIV, and HBV with the hope of supporting the discovery of new and alternative antiviral drugs.

## Method

### Search Scheme

Renowned and globally accepted scientific databases including Google scholar (https://scholar.google.com/), PubMed (https://pubmed.ncbi.nlm.nih.gov/), ScienceDirect (http://www.sciencedirect.com/), Scopus (http://www.scopus.com/), Springer Link (http://link.springer.com/), and Wiley Online Library (http://onlinelibrary.wiley.com/) were accessed to search literatures by emphasizing specific terminologies, such as “antiviral,” “medicinal plants,” “Bangladesh,” “Indian subcontinent,” “bioactive compounds,” “structure activity relationship,” and “antiviral mechanism”. Only literatures written in English language were considered due to language barrier.

### Inclusion Criteria and Data Extraction

In this review, studies covering following types of data were included and extracted: medicinal plants with antiviral activity along with their distribution, availability, traditional and folklore use, *in vitro* and *in vivo* studies of plant extracts and isolated bioactive compounds, their structural activity relationship and mechanism of antiviral activities. The focus of this review was on potential antiviral metabolites indigenous to and cultivated in Bangladesh. Due to lack of adequate scientific data regarding antiviral activities of medicinal plants collected from Bangladesh, available studies conducted on similar plant species in different countries are considered.

## Antiviral Plants of Bangladesh

In this review, we have discussed the antiviral activities of medicinal plants indigenous to and/or cultivated in Bangladesh along with their phytocompounds and the corresponding mechanisms of antiviral activity. A total of 46 antiviral plants from 25 families were substantiated in Table 1. According to families, medicinal plants were categorized. About 36 bioactive metabolites with significant effects and their underlying mechanisms of these antiviral activities were summarized in Table 3.

**TABLE 1:** Overview of the effects of medicinal plants extracts on common viral infections.

Family	Species	Extract type	Part used	Bioactive compound	Antiviral activity	References
*Acanthaceae*	*Acanthus ilicifolius* L.	Alcoholic extract	Whole plant	—	HBV	Wei et al. (2015)
	*Andrographis paniculata* (Burm.f.) Nees	EE	Leaf	Andrographolide	HSV-I, HIV, and EBV	Jayakumar et al. (2013)
	*Justicia adhatoda* L.	ME	Leaf	Anisotine	SARS-CoV-2, influenza virus, and HSV	Chavan and Chowdhary (2014); Ghosh et al. (2021)
*Amaranthaceae*	*Achyranthes aspera L.*	ME	Leaf	Oleanolic acid	HSV-I and II	Mukherjee et al. (2013)
*Amaryllidaceae*	*Allium sativum* L.	AE, ME, EE, and n-hexane extract, and garlic oil	Bulb	Ajoene, allicin, alliin, allyl methyl thiosulfinate, methyl allyl thiosulfinat, allitridin, diallyl sulfide, garlicin, and lectin	ADV-3, ADV-41, DENV, SARS-CoV-2, HSV-I and II, HCMV, H9N2, IBV, H1N1, CBV-3, ECHO, EV-71, HRV-2, HAV, MeV, PIV-3, VV, VSV, HIV-1, REV	Rouf et al. (2020)
*Anacardiaceae*	*Mangifera indica* L*.*	AE	Fruit	Mangiferin	Human influenza virus, HSV-I, and HIV	Al-Rawi et al. (2019)
*Apocynaceae*	*Alstonia scholaris* (L.) R. Br.	EE fraction of total alkaloid	Leaf	Total alkaloid	IAV	Zhou et al. (2020)
	*Calotropis gigantea* (L.) Dryand.	—	Latex	(+)-pinoresinol 4-*O*-(6″-*O*-vanilloyl)-*β*-d-glucopyranoside 6′-*O*-vanilloyltachioside 6′-*O*-vanilloyl-isotachioside	Influenza (H_1_N_1_)	Parhira et al. (2014)
*Asphodelaceae*	*Aloe vera* (L.) Burm.f.	EE	Leaf	Feralolide, 9-dihydroxyl-2-O-(z)-cinnamoyl-7-methoxy-aloesin, aloeresin, quercetin, catechin hydrate, and kaempferol	SARS-CoV-2, and influenza virus (H_1_N_1_ or H_3_N_2_)	Choi et al. (2019); Mpiana et al. (2020)
*Asteraceae*	*Eclipta prostrata L.*	ME	Leaf	Coumestan	HCV	Kaushik-Basu et al. (2008)
*Bombacaceae*	*Bombax ceiba L.*	EE	Flower	Kaempferol-3-*O*-(6″-*O*-*E*-*p*-coumaroyl)-β-d-glucopyranoside	RSV, and SARS-CoV-2	Schwarz et al. (2014); Zhang et al. (2015)
*Combretaceae*	*Anogeissus acuminata (Roxb. ex DC.) Wall. ex Guillem. & Perr*	—	—	Anolignan A Anolignan B	HIV	El-Ansari et al. (2020)
*Cyperaceae*	*Cyperus rotundus L.*	Essential oil	Rhizome	Humulene epoxide, and caryophyllene oxide	SARS-CoV-2, HAV, HSV-I, and CVB	Samra et al. (2020); Amparo et al. (2021)
*Fabaceae*	*Albizia procera* (Roxb.) Benth.	EE, and EAE	Bark	(+)-catechin, and protocatechuic acid	IAV	Panthong et al. (2015)
	*Butea monosperma* (Lam.) Taub.	AE	Bark, flower, fruit, leaf, and root	5,7-dihydroxy -3,6,4-trimethoxy flavone-7-O-α-L xylopyranosyl (1→3)-O-α-L arabinopyranosyl-(1→4)-O-β-D galactopyranoside	EV-71	Panda et al. (2017); Tiwari et al. (2019)
*Flacourtiaceae*	*Flacourtia indica* (Burm.f.) Merr.	EAE	Stem bark	Flacourtosides A and E, betulinic acid 3β-caffeate, and scolochinenoside D	DENV, and CHIKV	Bourjot et al. (2012)
*Gentianaceae*	*Swertia angustifolia* var. pulchella (D. Don) Burkill	—	Whole plant	(+)-cycloolivil-4′-O-β-d-glucopyranoside, swertiachiralatone A, swertiachoside A, swertiachirdiol A, and swertiachoside B	HBV, and HSV-I	Verma et al. (2008); Zhou et al. (2015)
*Lamiaceae*	*Ocimum tenuiflorum* L.	AE, and EE	Aerial part	Ursolic acid, Eugenol, 1,8- cineole and, rosmarinic acid	HSV-I, and II	Caamal-Herrera et al. (2016)
	*Ocimum basilicum* L.	ME, and EE	Aerial part	1,8-cineole, camphor, thymol, eugenol, eugenol epoxide, apigenin, linalool, and ursolic acid	HIV-I, HSV, ADV-3, 8, 11, HVB, EV, and CVB-I	Behbahani et al. (2013); Kubiça et al. (2014); Tshilanda et al. (2020)
	*Ocimum gratissimum* L.	Essential oil	Leaf	Eugenol, and thymol	HSV-I, and II	Maria das Graças et al. (2007); Benencia and Courreges (2000); Benitez et al. (2009); Lai et al. (2012)
	*Ocimum campechianum* Mill.	Essential oil, and AE	Leaf, Aerial part	β-caryophyllene, and 1,8-cineole	HSV-I, II, and IBV	Maria das Graças et al. (2007); Astani et al. (2010); Yang et al. (2010); Tshilanda et al. (2020)
	*Ocimum americanum* L.	ME, and DE	Leaf	Rosmarinic, and oleanolic acid	EV-71, and HIV-I	Aluko et al. (2012); Chung et al. (2015); Pandey et al. (2017); Tshilanda et al. (2019)
	*Ocimum × africanum* Lour.	EE	Leaf, aerial part	Caffeic acid, and linalool	HSV-I, and ADV-11	Romeilah et al. (2010); Ikeda et al. (2011); Pandey et al. (2017)
	*Ocimum forsskaolii* Benth.	EE	Leaf	Ursolic acid	HCV	Silva et al. (2008)
	*Ocimum carnosum (Spreng.) Link & Otto ex Benth.*	Essential oil	Leaf	Trans-anethole	HSV-I, and II	Astani et al. (2010)
*Meliaceae*	*Azadirachta indica* A.Juss.	AE	Bark and leaf	Gedunin, pongamol, and azadirachtin	HSV-I, CVB-B4, HBV, and SARS-CoV-2	Alzohairy (2016); Rao and Yeturu (2020); Nesari et al. (2021)
	*Melia azedarach L.*	EAE	Leaf	Limonoid 1-cinnamoyl-3,11-dihydroxymeliacarpin	VSV, and HSV-I	Alché et al. (2003)
*Moraceae*	*Ficus religiosa L.*	ME, AE, and chloroform extracts	Bark	—	RSV, HRV, and HSV-II	Cagno et al. (2015); Ghosh et al. (2016)
	*Artocarpus integer* (Thunb.) Merr.	AE	Bark	—	Rotavirus	Gonçalves et al. (2005)
	*Artocarpus heterophyllus* Lam.	DE	Leaf	—	HCV	Hafid et al. (2017)
	*Artocarpus camansi* Blanco	DE	Leaf	—	HCV	Hafid et al. (2017)
	*Artocarpus altilis* (Parkinson ex F.A.Zorn) Fosberg	DE	Leaf	—	HCV	Hafid et al. (2017)
*Phyllanthaceae*	*Phyllanthus niruri* L.	AE, and EE	Whole plant	Phyllanthin, and hypophyllantin	HBV, WHV, and HCV	Tan et al. (2013); Wahyuni et al. (2019)
*Piperaceae*	*Piper longum* L.	EE	Seed	Piperine	VSV-IN, PIV, and HBV	Jiang et al. (2013); Priya and Saravana Kumari (2017)
	*Piper nigrum* L.	—	Seed	Guaiol	VSV-IN, PIV, and SARS-CoV-2	Pandey et al. (2021) Priya and Saravana Kumari (2017)
*Poaceae*	*Cynodon dactylon* L.	—	Whole plant	—	BCoV	Nalanagula, (2020)
*Rosaceae*	*Rosa centifolia* L.	ME	Leaf	—	HIV	Palshetkar et al. (2020)
*Rubiaceae*	*Hedyotis scandens* Roxb.	EE	Whole plant	Maltol 60-b-D-apiofuranosyl-b-D-gluco-pyranoside, and grevilloside G	RSV	Wang et al. (2013)
*Rutaceae*	*Aegle marmelos* (L.) Corrêa	—	—	Seselin	SARS-CoV-2	Nivetha et al. (2021)
	*Citrus limon* (L.) Osbeck	Essential oil	Fruit	Luteolin	HAV	Battistini et al. (2019)
	*Citrus sinensis* (L.) Osbeck	Essential oil	Fruit	Hesperidin, luteolin, and vitamin C	HAV, and SARS-CoV-2	Battistini et al. (2019); Bellavite and Donzelli (2020)
	*Citrus paradisi* Macfad.	Essential oil	Fruit	—	HAV	Battistini et al. (2019)
*Theaceae*	*Camellia sinensis* (L.) Kuntze	—	Leaf	Epigallocatechin-3-gallate (EGCG), epicatechin gallate (ECG), epicatechin (EC), and catechin	HIV, HSV-I, IAV, HCV, HBV, VSV, reovirus, mCMV, DENV, JEV, CHIKV, ZIKV, TBEV, EV71, and rotavirus	Xu et al. (2017)
*Urticaceae*	*Boehmeria nivea* L.	EE	Root	—	HBV	Chang et al. (2010)
*Zingiberaceae*	*Zingiber officinale* Roscoe	AE	Rhizome	6-gingerol, and gingeronone A	CHIKV, HCV, and SARS-CoV-2	Pandey et al. (2021); Abd El-Wahab et al. (2009); Kaushik et al. (2020); Rathinavel et al. (2020)
	*Curcuma longa* L.	AE	Rhizome	Curcumin	HBV, SARS-CoV-2, HIV, IAV, DENV, CHIKV, VSV, ZIKV, Kaposi sarcoma-associated HSV, and RSV	Kim et al. (2009); Jennings and Parks (2020); Thimmulappa et al. (2021)

— indicates not found; AE, aqueous extract; ME, methanolic extract; EE, ethanolic extract; DE, dichloromethane extract, and EAE, ethyl acetate extract.

### Acanthaceae

*Acanthus ilicifolius* L. belonging to family Acanthaceae, is a mangrove plant with numerous medicinal properties, including anti-inflammatory, antioxidant and hepatoprotective activities. This medicinal plant exhibits potent antiviral activity against hepatitis B virus. A study performed on duck model revealed that alcoholic extract of whole plant is capable of reducing the viral load by interfering DNA replication, but the exact mechanism was not explained well (Wei et al., 2015). *Andrographis paniculata* (Burm.f.) Nees belongs to Acanthaceae family as well. It possesses excellent neutralizing activity against the human immunodeficiency virus (HIV). Andrographolide is a phytochemical isolated from this plant which has been reported for antiviral activity against herpes simplex virus (HSV), HIV, flaviviruses, and pestiviruses (Jayakumar et al., 2013). This compound inhibited HIV-induced cell cycle dysregulation which results the increase of CD4^+^ lymphocyte levels in HIV-1 infected people (Calabrese et al., 2000). Besides, this bioactive compound has been reported for inhibition of the expressions of HSV-I viral envelope glycoproteins D and C (Wiart et al., 2005). Another study revealed that ethanolic extract (25 μg/ml) of *A. paniculata* as well as andrographolide (5 μg/ml) remarkably inhibited the expression of Epstein-Barr virus (EBV) lytic proteins, Rta, Zta, and EA-D in the viral lytic cycle in P3HR1 cells (Lin et al., 2008). This study has also demonstrated that andrographolide is not-toxic to P3HR1 cells at a dose of <5 μg/ml. This compound is now under clinical trial (phase-IV) for treatment of bronchitis (Table 2).

**TABLE 2:** Plant metabolites studying under clinical trial as antiviral agents.

Intervention	Phase	Indication	Primary purpose	Study place	References
Andrographolide	IV	Acute Bronchitis	Treatment	China	https://clinicaltrials.gov/ct2/show/NCT03132623
Quercetin	N/A	COVID-19	Prevention	Turkey	https://clinicaltrials.gov/ct2/show/NCT04377789
Hesperidin	II	COVID-19	Treatment	Canada	https://clinicaltrials.gov/ct2/show/NCT04715932
Curcumin	N/A	COVID-19	Prophylaxis	India	http://www.ctri.nic.in/Clinicaltrials/pdf_generate.php?trialid=45936&EncHid=&modid=&compid=%27,%2745936det%27

*Justicia adhatoda* L. is another member of Acanthaceae family which is native to Bangladesh. It is known as malabar nut, adhatoda or vasaka and traditionally used in cold, cough and respiratory disorders from ancient times. Methanolic extract of the leaves of this medicinal plant has been reported for inhibitory activities against influenza and herpes simplex virus (HSV). Six alkaloids namely vasicoline, vasicolinone, vasicinone, vasicine, adhatodine and anisotine have been isolated from the leaves of *J. adhatoda*. *In silico* bioassay demonstrated that anisotine has significantly inhibited the main protease (Mpro) of SARS-CoV-2. Mpro mediates the cleavage of polyprotein to get matured and acquire infectivity. The assay has also suggested that inhibitory potential of this alkaloid is higher compared to the inhibitory activities of lopinavir and darunavir (established antiviral drugs) (Ghosh et al., 2021).

### Amaranthaceae

*Achyranthes aspera* L. belonging to the family Amaranthaceae, is a medicinal plant of the Garo tribe population in the Madhupur forest region of Bangladesh. It is a well-known folk medicine not only in Bangladesh but also in Indian subcontinent. It contains a potent antiviral compound named oleanolic acid which has been reported to work against herpes simplex virus type-I, HSV-I (EC_50_ 6.8 μg/ml) and type-II, HSV-2 (EC_50_ 7.8 μg/ml) (Mukherjee et al., 2013). Both the plant extract and oleanolic acid inhibited the early stage of multiplication, specifically 2–6 h of post infection of the viruses.

### Amaryllidaceae

*Allium sativum* L., a species of Amaryllidaceae family is considered as one of the rich sources of medicinal substances and has been used for healing infectious diseases like cold, flu, asthma and other viral infections from ancient time in traditional Chinese medicine, Islamic medicine and folklore. In Bangladesh, it is cultivated all over the country as a fundamental spice used in cooking. A study has been documented that various extracts of *A. sativum* have inhibitory activities against adenovirus-3 (ADV-3), adenovirus-41 (ADV-41) (Khanal et al., 2018), dengue virus (DENV) (Alejandria, 2015), SARS-CoV-2 (Rouf et al., 2020), HSV-I and II (Straface et al., 2012), human cytomegalovirus (HCMV), influenza A virus (IAV) subtype H_1_N_1_ and H_9_N_2_, influenza B virus (IBV) (Mettenleiter and Sobrino, 2008), coxsackie B virus (CBV-3), echovirus-11 (ECHO), enterovirus (EV-71), human rhinovirus-2 (HRV-2), HAV, measles virus (MeV), parainfluenza virus-3 (PIV-3), vaccinia virus (VV), vesicular stomatitis virus (VSV), HIV-1 (Wang et al., 2017), and reticuloendotheliosis virus (REV). Numerous antiviral phytocompounds have been isolated from a number of extracts of the bulb of *A. sativum* including ajoene, allicin, alliin, allyl methyl thiosulfinate, allitridin, diallyl sulfide, garlicin, and lectins. Ajoene prevents HIV-induced destruction of CD4^+^ cells and enhances cellular immunity. It also inhibits viral attachment to host cell and reverse transcriptase of HIV-I. Apart from these, it induces apoptosis of HCMV infected cells. Allicin and allyl methyl thiosulfinate inhibit the entry of HSV-I and II, PIV-3, VV, VSV and HRV-2 by disrupting viral envelope and cell membrane. Moreover, allicin inhibits the replication of REV by downregulation of ERK/MAPK pathway. Alliin, diallyl sulfide, and garlicin work against DENV by diminishing inflammation through suppressing oxidative stress. Allitridin has excellent multiple effects against HCMV. The underlying mechanisms of these activities include inhibition of viral DNA synthesis by interfering viral immediate-early antigen expression, inhibition of viral replication by suppressing viral IEG gene transcription, and enhancement of Treg expansion and Treg-mediated anti-HCMV immunosuppression (Alejandria, 2015; Wang et al., 2017; Rouf et al., 2020).

### Anacardiaceae

*Mangifera indica* L. is one of the most common plants for fruit considering as the king of all fruits in Bangladesh. It belongs to the family Anacardiaceae. This fruit is packed of antioxidants and other nutritious biomolecules. The plant extract has been reported for its activity against influenza virus. Apart from this, it contains a bioactive compound named mangiferin having potential efficacy for inhibiting the duplication of HSV-I and antagonizing the cytopathic effects of HIV (Al-Rawi et al., 2019).

### Apocynaceae

*Alstonia scholaris* (L.) R. Br., a species of Apocynaceae family, is a folklore medicine in Bangladesh usually used to treat cold, cough, asthma, and chronic obstructive pulmonary disease (COPD). This plant is a rich source of total alkaloids having remarkable anti-inflammatory and antiviral activities. A study demonstrated that the total alkaloids present in this plant exhibited efficacy to fight against IAV. The mechanism of this antiviral activity involves inhibition of viral replication (in A549 cells and U937-derived macrophages), reduction of cytokine and chemokine generation at the mRNA and protein levels, as well as interfering the activation of pattern recognition receptor (PRR)- and IFN-activated signal transduction (in A549 cells). Along with these, increment of survival rate and reduction of the viral titer were observed in lethal PR8 mouse model (Zhou et al., 2020).

Another important species of Apocynaceae family is *Calotropis* gigantea (L.) Dryand., also known as milk weed which is found in Bandarban, Chattogram, Cox’s Bazar, Khagrachari, and Rangamati of Bangladesh. From the latex of the plant, a lignan glycoside namely (+)-pinoresinol 4-O-(6″-O-vanilloyl)-β-d-glucopyranoside and two phenolic compounds such as 6′-O-vanilloyltachioside and 6′-O-vanilloylisotachioside have been isolated. Among them, the lignin glycoside was efficacious against H_1_N_1_ strain of both of the subtypes A and B (IC_50_ value of 13.4–39.8 μg/ml). The demonstrated underlying mechanism of this activity involved inhibition of NF-κB pathway and viral ribonucleoproteins nuclear exporting without interfering virus-induced activation of Raf/MEK/ERK pathway (Parhira et al., 2014).

### Asphodelaceae

*Aloe vera* (L.) Burm.f. is a well-known medicinal plant belonging to Asphodelaceae family and found almost everywhere in Bangladesh. *A. vera* gel (0.2–5%) has been reported for inhibitory activity on HSV-I growth in Vero cell line. This study has demonstrated that the gel is effective as topical treatment option for oral HSV-I infection (Rezazadeh et al., 2016). An *in silico* study revealed that treatment with ethanolic extract of *A. vera* significantly reduces of the replication of IAV along with inhibition of viral matrix protein 1 (M1), matrix protein 2 (M2), and hemagglutinin (HA) mRNA synthesis, and expressions of viral protein (M1, M2, and HA). Numerous potent antiviral bioactive compounds, such as quercetin, catechin hydrate, and kaempferol were isolated which have inhibited IAV (H1N1 or H3N2) induced autophagy, M2 viral mRNA synthesis, and M2 protein expression. Apart from these, *in silico* docking simulation study stated that these bioactive compounds have higher binding affinity (for M2 protein) compared to established M2 protein inhibitors (Choi et al., 2019). Recently, COVID-19 pandemic has created worldwide burden because of the unavailability of the suitable medical treatment option. Quercetin is under clinical trial for prophylaxis as well as management of the symptoms of this infection (Table 2). Furthermore, *A. vera* has been reported to contain 9-dihydroxyl-2-O-(z)-cinnamoyl-7-methoxy-aloesin, aloeresin and feralolide which showed potential to inhibit the main protease (3CLpro) responsible for the replication of SARS-CoV-2 in an *in silico* investigation. This study also demonstrated that feralolide might be one of the foremost choices for development of potential drug for COVID-19 infection due to its higher binding affinity to 3CLpro, and drugability (according to the Lipinski’s rule of five) (Mpiana et al., 2020).

### Asteraceae

*Eclipta prostrata* L. is the only known member of Asteraceae family which has strong antiviral property. In Bangladesh, this valuable medicinal plant grows wildly in fallow lands and the cultivators consider them as weed. This plant is known as kalo keshi and used as folklore medicine to treat snake bite and blood borne hepatitis. Coumestan is a phytosterol found in this plant which has been reported for excellent inhibiting activity against NS5B protein of HCV. This protein is essential for viral RNA replication (Kaushik-Basu et al., 2008). Therefore, this compound and its analogs might be targeted for development of novel replication inhibitors of HCV.

### Bombacaceae

*Bombax ceiba* L., a member of Bombacaceae family, is very common plant in Bangladesh and found almost everywhere. It is also known as cotton tree because of producing cotton from flowers. Flower of this plant produces a flavonoid glycoside having a *cis*-coumaroyl connection, namely kaempferol-3-*O*-(6″-*O*-*E*-*p*-coumaroyl)-β-d-glucopyranoside. This flavonoid glycoside has been reported for having inhibitory activity on respiratory syncytial virus (RSV) (Zhang et al., 2015). Besides, an *in silico* study stated that Kaempferol-3-*O*-(6″-*O*-*E*-*p*-coumaroyl)-β-d-glucopyranoside inhibits the open-reading-frame 3a (ORF 3a) protein of SARS-CoV-2. This protein is crucial for expression of a cation-selective channel which regulates viral release mechanism (Schwarz et al., 2014).

### Combretaceae

*Anogeissus acuminata (Roxb. ex DC.) Wall. ex Guillem. & Perr.* is an Asian species of Combretaceae family which is found in Bandarban, Chattogram, Cox’s Bazar, Khagrachari and Rangamati area of Bangladesh. This plant produces two dibenzylbutadiene lignans, namely anolignan A and anolignan B which showed significant inhibitory activity against HIV-I reverse transcriptase (RT) enzyme. Besides, both of the phytocompounds exerted a synergistic activity against this enzyme (El-Ansari et al., 2020).

### Cyperaceae

*Cyperus rotundus* L. belonging to family Cyperaceae, is considered as a troublesome and economically damaging weed found in almost all the croplands in Bangladesh. Surprisingly, this plant has numerous medicinal properties, including antidiarrheal, antioxidant, anti-inflammatory, antimutagenic, antiperiodic, anticonvulsant, anti-saturative, antipyretic, antifungal, antidiabetic, antimalarial, antilipidemic, antibacterial, antiviral, anti-tumoral, cardioprotective, and wound-healing properties (Peerzada et al., 2015). A study demonstrated that essential oil extracted from the rhizomes of this plant has inhibitory activity against HAV, HSV-I, and CVB. Humulene epoxide and caryophyllene oxide were identified as major bioactive compounds from this essential oil (Samra et al., 2020). Caryophyllene oxide has been reported to exhibit very potent inhibitory activity against HSV-I which might be a prime lead for development of topical therapeutic agent to treat recurrent infection caused by HSV-I (Astani et al., 2011). Moreover, an *in silico* study demonstrated that humulene epoxide has remarkable binding affinity to four target proteins, such as spike glycoprotein, papain-like protease (PLpro), 3-chymotrypsin-like protease (3CLpro), and RNA-dependent RNA polymerase (RdRp) which are crucial for regulation of lifecycle of SARS-CoV-2 (Amparo et al., 2021).

### Fabaceae

*Albizia procera* (Roxb.) Benth., a member of Fabaceae family is found in forests of Chittagong, Chittagong Hill Tracts, Cox’s Bazar, and Dhaka-Mymensingh Sal forests of Bangladesh. It is very popular traditional medicinal plant whose bark (decoction) is used to manage rheumatism, hemorrhage, and stomach-ache (Sivakrishnan and Swamivelmanickam, 2019). This plant has potent antiviral activity against IAV. A study showed that ethanolic, ethyl acetate, aqueous and hexane-chloroform extracts of the bark of *A. procera* have inhibited the integrase enzyme of IVA with IC_50_ value of 19.5, 19.1, 21.3,  and  >100  μg/ml respectively. Two major compounds such as (+)-catechin and protocatechuic acid have been isolated from the bark of this plant. (+)-Catechin showed substantial activity against IAV intergase (IC_50_ value: 46.3 µM), whereas the effect of protocatechuic acid was mild. *In silico* docking study suggested that (+)-catechin interacts with Thr66, Gly148, and Glu152 in the core domain of integrase enzyme, whereas protocatechuic acid binds to Thr66, His67, Glu152, Asn155, and Lys159 (Panthong et al., 2015).

*Butea monosperma* (Lam.) Taub., another important member of Fabaceae family, is a well-known medicinal plant found in almost everywhere in Bangladesh and known as flame of forest (local name: Palash). In Ayurvedic, Unani and Homeopathic medicine, this plant has numerous medicinal uses. However, scientific literature demonstrated that aqueous extract of various parts of this plant like bark, flowers, fruit, leaves, and roots showed significant inhibition of EV-71 (BrCr) (Panda et al., 2017). A flavone glycoside, namely 5,7-dihydroxy-3,6,4-trimethoxy flavone-7-O-α-L xylopyranosyl (1→3)-O-α-L arabinopyranosyl-(1→4)-O-β-D galactopyranoside has been isolated form the flower of this plant which showed significant antiviral activity (Tiwari et al., 2019).

### Flacourtiaceae

*Flacourtia indica* (Burm.f.) Merr. is a tropical species of family Flacourtiaceae with broad geographical distributions covering Bangladesh. It is an edible wild fruit species used by the traditional medical practitioners for treating snakebite. This medicinal plant has been reported for inhibitory activity against chikungunya (CHIKV) and dengue (DENV) viruses. Ethyl acetate extract of stem bark of this plant has inhibited CHIKV. Moreover, significant inhibitory activity has been observed against DENV RNA polymerase enzyme by the isolated compounds, such as flacourtosides A and E, betulinic acid 3β-caffeate (IC_50_ = 0.85 ± 0.1 μM), and scolochinenoside D (IC_50_ values ∼10 μM) (Bourjot et al., 2012).

### Gentianaceae

*Swertia angustifolia var. pulchella (D. Don) Burkill* belonging to the family Gentianaceae, is a medicinal plant of Bangladesh which is mainly distributed in the mountainous regions. It is known as Ayurvedic herb and is usually used to treat malaria and diabetes. Besides, local populations use this herb as folklore medicine to manage hepatitis, inflammation, and digestive disorders. Crude extract of this herb has been reported for exhibiting activity against HSV-I (Verma et al., 2008). A novel bioactive compound named (+)-cycloolivil-4′-O-β-d-glucopyranoside has been isolated from this herb which inhibited HBsAg and HBeAg secretion (IC_50_ values: 0.31 ± 0.045 mM and 0.77 ± 0.076 mM respectively) as well as HBV DNA replication (IC_50_ value: 0.29 ± 0.034 mM) in anti-HBV assay on HepG 2.2.15 cells line (Zhou et al., 2015).

### Lamiaceae

The genus, *Ocimum* is a broad member of Lamiaceae family which are found everywhere in Bangladesh and known as “the medicinal herb for all disease”. Species of this genus exhibit numerous medicinal properties and have been used from ancient time as folklore medicines. The genus is actually the biggest sources of antiviral phytocompounds (Tshilanda et al., 2020). About 8 species of this genus are found in Bangladesh, namely *Ocimum tenuiflorum* L., *Ocimum basilicum* L., *Ocimum gratissimum* L., *Ocimum campechianum* Mill., *Ocimum americanum* L., *Ocimum × africanum* Lour., *Ocimum forsskaolii* Benth., *and Ocimum carnosum* (*Spreng.*) *Link & Otto ex Benth.* which have been reported extensively for diverse antiviral activities.

*O. tenuiflorum* is commonly known as “basil or holy basil” which is considered as holy plant according to Hinduism. This medicinal plant is found almost every yard of people in Bangladesh. It produces a number of antiviral bioactive compounds, such as ursolic acid, eugenol, 1,8-cineole, and rosmarinic acid which exhibit potential to inhibit HSV-I and II (Caamal-Herrera et al., 2016). *O. basilicum*, known as sweet basil, contains 1,8-cineole, camphor, thymol, eugenol, eugenol epoxide, apigenin, linalool, and ursolic acid which have been reported to work against HIV-I, HSV, ADV-3, 8, 11, HVB, EV, and CVB-I (Behbahani et al., 2013; Kubiça et al., 2014; Tshilanda et al., 2020). *O. gratissimum* is an aromatic herb which is commonly known as African basil. Essential oil of this basil leaves contains two alcohols namely eugenol and thymol. Eugenol inhibits replication of HSV-I and II while thymol destructs the virion of HSV-I (Benencia and Courreges, 2000; Maria das Graças et al., 2007; Benitez et al., 2009; Lai et al., 2012). β-caryophyllene and 1,8-cineole have been isolated from *O. campechianum* which exhibit anti-HSV-I and II activities as well as inhibit infectious bronchitis virus (IBV) (Maria das Graças et al., 2007; Astani et al., 2010; Yang et al., 2010; Tshilanda et al., 2020). *O. americanum*, recognized as American basil, is a medicinal plant which can produce essential oils and found in Bangladesh. Rosmarinic acid and oleanolic acid are the essential oils isolated from this herb. Oleanolic acid inhibits HIV-I protease whereas rosmarinic acid inhibits internal ribosome entry site of EV-71 (Aluko et al., 2012; Chung et al., 2015; Pandey et al., 2017; Tshilanda et al., 2019). *O. africanum* produces caffeic acid which inhibits the multiplication HSV-I. Beside, linalool has also been isolated from the essential oil of this medicinal plant havinganti-ADV-11 activity (Romeilah et al., 2010; Ikeda et al., 2011; Pandey et al., 2017). *O. forsskaolii* is known as wild Amazonian basil which produces ursolic acid which exhibits anti-HCV activity. Moreover, *O. carnosum* showed anti-HSV-I and II activities due to presence of trans-anethole which inhibits multiplication of HSV-I and II (Tshilanda et al., 2020).

### Meliaceae

*Azadirachta indica* A. Juss., commonly known as “neem”, is a member of Meliaceae family which is found almost everywhere in Bangladesh. This medicinal plant has a lot of medicinal properties and so, has been used for health management from ancient time in folklore, Ayurvedic, and Unani medicinal systems. At a therapeutic concentration of 50–100  μg/ml, aqueous extract of *A. indica* bark remarkably blocked the entry of HSV-I into host cells. Virucidal activity against CVB-B4 was observed by the extract of *A. indica* leaves (Alzohairy, 2016). Gedunin and pongamol are the antiviral biocompounds extracted from *A. indica* having activity against DENV. Gedunin showed significant binding affinity to NS3 RNA polymerase and NS3 protease helicase (mediate the synthesis of DENV proteins and genetic materials in the host cell) as well as capsid and envelope proteins (required for entry of DENV into host cells) (Rao and Yeturu, 2020). Moreover, “neem capsule” is under clinical trial for prophylaxis and prevention of COVID-19 infection (Nesari et al., 2021). *Melia azedarach* L. is another antiviral medicinal plant from Meliaceae family which has been reported for inhibitory activities against vesicular stomatitis (VSV) and HSV-I. A meliacarpin named limonoid 1-cinnamoyl-3,11-dihydroxymeliacarpin has been isolated from ethyl acetate extract of the leaves of this plant which showed inhibitory activities against VSV (IC_50_ values of 6 μM) and HSV-1 (IC_50_ values of 20 μM) (Alché et al., 2003).

### Moraceae

*Ficus religiosa* L. belongs to the family Moraceae which is used in traditional Ayurvedic and Unani medicines for healing cough, wheezing and asthma as well as sexually transmitted infections like gonorrhea and genital ulcers. This medicinal plant exhibits numerous antiviral activities. A study demonstrated that ethanolic extract of the bark of *F. religiosa* inhibited Human rhinoviruses (HRV) (EC_50_ value: 5.52 μg/ml) by interfering the late steps of replicative cycle. Aqueous extract showed inhibitory activity against respiratory syncytial virus (RSV) (EC_50_ value: 2.23–4.37 μg/ml) by partial inactivation as well as interfering attachment to host cells (Cagno et al., 2015). Another study stated that aqueous and chloroform extracts of bark were active against HSV-II and acyclovir-resistant strain. The underlying mechanism of the aqueous extract involved direct inactivation of viral activity whereas chloroform extract suppressed the attachment and entry of virus to host cell membrane along with inhibition of viral progeny formation (Ghosh et al., 2016).

Artocarpus genus is another source of antiviral medicinal plant species. *Artocarpus integer* (Thunb.) Merr., *Artocarpus heterophyllus* Lam., *Artocarpus camansi* Blanco and *Artocarpus altilis* (Parkinson ex F.A.Zorn) Fosberg are the species of this genus which have antiviral activity. A. *integer* has been reported to have activity against rotavirus (simian rotavirus, SA11 and human rotavirus, HCR3 strains) (Gonçalves et al., 2005). Another study showed that dichloromethane extract of the leaves of *A. heterophyllus* showed strong anti-HCV (IC_50_ value: 1.5 ± 0.6 μg/ml) without major toxicity, whereas that of *A. altilis* and *A. camansi* showed moderate anti-HCV activities (IC_50_ values: 6.5 ± 0.3 and 9.7 ± 1.1 μg/ml respectively). The underlying mechanism of such potent anti-HCV activity of *A. heterophyllus* involved synergistic effects such as direct virucidal activity (inhibition of viral entry) and inhibition of replication of RNA and expression of viral protein at higher concentration (Hafid et al., 2017).

### Phyllanthaceae

*Phyllanthus niruri* L., a member of Phyllanthaceae family is a medicinal plant found in Bangladesh which is used traditionally for management of edema, constipation, helminthiasis, dysentery, diarrhea, and pain. This plant possesses antiviral activity as well. Aqueous extract of whole plant has been reported to inhibit endogenous DNA polymerase of HBV and woodchuck hepatitis virus (WHV) (Tan et al., 2013). Another study stated that ethanolic extract of *P. niruri* has Anti-HCV activity (IC_50_ value: 4.14 μg/ml). Apart from this, it showed synergistic activity (4-fold) with an established drug, a NS3 protease inhibitor named simeprevir. Phyllanthin and hypophyllantin have been identified from this plant which showed binding to a protein, 4GAG required for entry of HCV to host cells in a *in silico* molecular docking assay (Wahyuni et al., 2019).

### Piperaceae

*Piper longum* L. and *Piper nigrum* L. are the two most common species of Piperaceae family which are cultivated in Bangladesh as spices of cooking. Both of these species exhibit a number of medicinal properties and thus, are used as folklore and traditional medicines from primordial times. Seeds of these medicinal plants have been reported for inhibitory activities on vesicular stomatitis indiana virus (VSV-IN) and human para influenza virus (PIV) (Priya and Saravana Kumari, 2017). *P. longum* contains piperine which is a potent anti-HBV compound functioning against the secretion of HBsAg (Selectivity Index, SI: 15.7) and HBeAg (SI: 16.8) (Jiang et al., 2013). Furthermore, *P. nigrum* contains guaiol which has been reported by an *in silico* study to possess inhibitory potential to 6LU7 and 7JTL (crucial targets of coronavirus) (Pandey et al.).

### Poaceae

*Cynodon dactylon* L. is a non-toxic and edible grass belonging to Poaceae family which is known as durva grass or Bermuda grass It is found all over the countryside of Bangladesh and used as expectorant, emetic, laxative, coolant, analgesic, aphrodisiac, alexipharmic, emmenagogue, and so others. This medicinal plant is very effective against bovine coronavirus infection (BCoV) which functions by inhibiting protease enzyme. As this viral strain has some common features with SARS-CoV and SARS-CoV-2, it can be used as dietary intervention of COVID-19 (Nalanagula, 2020).

### Rosaceae

*Rosa centifolia* L., a flowering plant of Rosaceae family, is found in Bangladesh and known as Cabbage rose. The leave of this plant has antiviral activity. Methanolic extract of the leaves of *R. centifolia* L showed anti-HIV activity (Palshetkar et al., 2020).

### Rubiaceae

*Hedyotis scandens* Roxb. is a medicinal plant of Rubiaceae family found in tribal hill area of Bangladesh. This plant is used as folklore medicine in Chakma tribe. Two antiviral bioactive compounds have been isolated from ethanolic extract of the whole plant namely maltol 60-b-D-apiofuranosyl-b-D-glucopyranoside, and grevilloside G. These phytocompounds showed anti-RSV activity. IC_50_ values for these compounds were 20 and 25 μg/ml respectively (Wang et al., 2013).

### Rutaceae

*Aegle marmelos* (L.) Corrêa, a member of Rutaceae family, is a food producing plant which is found everywhere in Bangladesh. It is commonly known as bael or stone apple or wood apple. In Ayurveda, various parts of this plant are used because of having antidiarrhoeal, antimicrobial, antiviral, radioprotective, anticancer, chemopreventive, antipyretic, ulcer healing, antigenotoxic, diuretic, antifertility, and anti-inflammatory properties. This plant produces a bioactive compound named seselin having activity against multiple targets of SARS-CoV-2. *In silico* molecular docking study showed that seselin has inhibitory potential to the receptors SARS-CoV-2S protein (binding energy: 6.6 kcal/mol), COVID-19 main protease (−6.9 kcal/mol), and free enzyme of the SARS-CoV-2 (2019-nCoV) main protease (−6.7 kcal/mol) (Nivetha et al., 2021).

A number of citrus fruits producing medicinal plants namely *Citrus limon* (L.) Osbeck, *Citrus sinensis* (L.) Osbeck, and *Citrus paradisi* Macfad. are also found in this family which are commonly known as lemon, orange and grapefruit sequentially. All of them are very rich sources of vitamin C which fastens healing of COVID-19 by boosting immunity (Bellavite and Donzelli, 2020). Essential oils extracted from the fruits of these medicinal plants have been reported for having inhibitory potential to HAV (Battistini et al., 2019). Potent antiviral compounds named hesperidin and luteolin have been isolated from fruit of *C. sinensis.* An *in silico* study has demonstrated that hesperidin showed efficacy to inhibit spike protein and Mpro that modulate the immature proteins (pp1a and ppa1b) to the complex and functional one to progress replication process of SARS-CoV-2 (Bellavite and Donzelli, 2020). Furthermore, luteolin has also been reported for having inhibitory activities against ACE2 receptor (both of the subtypes AT1 and AT2) and RdRp enzyme by an *in silico* assay (Goyal et al., 2020).

### Theaceae

*Camellia sinensis* (L.) Kuntze belonging to the family Theaceae is known as tea or green tea which is considered as the most popular drink in worldwide. In Bangladesh, this plant is cultivated in two fairly divergent ecological zones such as Surma valley in greater Sylhet and Halda valley in Chittagong (Mamun, 2019). The novel antiviral bioactive compounds namely epigallocatechin-3-gallate (EGCG), epicatechin gallate (ECG) and epicatechin (EC) have been isolated from the leaves of this plant. EGCG has been reported for surprising and divergent antiviral activities. It binds to virion surface proteins and blocks the attachment of HSV-I to heparan sulfate of host cells. It inhibits RNA and DNA synthesis as well as antigen expression in HBV. It has broad-spectrum antiviral activities on HCV, IAV, murine cytomegalovirus (mCMV), vesicular stomatitis virus (VSV), and reovirus as well. Apart from these, EGCG showed potency to inhibit HIV reverse transcriptase by downregulation of the expression of the HIV p24 antigen. A destructive effect has been observed on HIV-I viral particle. It interferes with HIV-I attachment to host cell surface too. Moreover, DENV, Japanese encephalitis virus (JEV), tick-borne encephalitis virus (TBEV), Zika virus (ZIKV), CHIKV, EV-71, and rotaviruses are also inhibited by EGCG (Xu et al., 2017).

### Urticaceae

*Boehmeria nivea* L. is the only species of Urticaceae family which exhibits antiviral activity. It is found in Bandarban, Khagrachari and Rangamati area of Bangladesh and traditionally used to prevent miscarriage as well as promote the drainage of pus and healing of wound and infections. A study demonstrated that ethanolic extract of the root exhibits anti-HBV activity. The possible mechanism suggested by the author involved potential inhibition of the expression of HBsAg and DNA of HBV (Chang et al., 2010).

### Zingiberaceae

*Zingiber officinale* Roscoe belongs to the family Zingiberaceae which is commonly known as ginger and cultivated in Bangladesh as a prime spice of cooking. In Ayurveda, the rhizome of this herb is used from pre-historic time because of having anti-arthritis, anti-inflammatory, antidiabetic, antibacterial, antifungal, and anticancer properties. Aqueous extract prepared from the freeze dried powder of the rhizome of this herb showed anti-HCV and anti-CHIKV activities. Active metabolites gingeronone A and 6-gingerol, isolated from the rhizome of *Z. officinale* have been reported for having anti-SARS-CoV-2 activity in molecular docking studies. Besides, 6-gingerol exhibits efficacy to inhibit SARS CoV-2 by interacting viral proteases, RNA binding protein, and Spike protein (Rathinavel et al., 2020). On the other hand, gingeronone A inhibits main protease (6LU7) and SARS-CoV-2 ORF8 (7JTL) (Pandey et al., 2021).

*Curcuma longa* L. is another species of Zingiberaceae family having numerous medicinal properties. It is also a spice used as foodstuff and cultivated in Bangladesh. It is used as herbal medicine for managing rheumatoid arthritis, chronic anterior uveitis, conjunctivitis, skin cancer, small pox, chicken pox, wound healing, urinary tract infection, and cancers. Aqueous extract of the rhizome of this herb has anti-HBV activity. It blocked HBx gene transcription by suppressing HBV enhancer I and X promoter through p53 protein (Kim et al., 2009). This herb produces curcumin which possesses diverse pharmacological activities. It inhibits HIV, DENV, CHIKV, ZIKV, VSV, IAV, RSV, EV71 and Kaposi’s sarcoma-associated herpesvirus by multiple pathways (Jennings and Parks, 2020) described in Table 3. Furthermore, a randomized controlled trial has proved the effectiveness of curcumin for pre-exposure prophylaxis of COVID-19 (Table 2). This prophylactic activity may be due to (a) multiple antiviral mechanisms of action (interact directly with viral membrane proteins, disrupt viral envelope, inhibit viral protease, and induce host antiviral response by boosting immunity) against numerous types of enveloped viruses (as SARS-CoV-2 is a enveloped virus) (b) protection from severe pneumonia (by targeting NF-κB, IL-6 trans signal, and HMGB1 pathways), and (c) safe and well-tolerated in both healthy and diseased human subjects (Thimmulappa et al., 2021).

**TABLE 3:** Bioactive compounds with antiviral mechanism isolated from medicinal plants.

Bioactive compounds	Plants	Mechanism of antiviral activity	References
Andrographolide	*Andrographis paniculata* (Burm.f.) Nees	a) Inhibit the expression of HSV-I enveloped glycoproteins D and C	Calabrese et al. (2000); Wiart et al. (2005); Lin et al. (2008)
		b) Inhibit HIV-induced cell cycle dysregulation and increase CD4^+^ lymphocyte	
		c) Inhibit the expression of EBV lytic proteins, Rta, Zta and EA-D	
Anisotine	*Justicia adhatoda* L.	Inhibit Mpro of SARS-CoV-2 which mediates the cleavage of polyprotein to get matured and acquire infectivity	Ghosh et al. (2021)
Oleanolic acid	*Achyranthes aspera* L. *Ocimum americanum* L.	a) Inhibited the early stage of multiplication (2–6 h of post infection) of HIV	Mukherjee et al. (2013); Tshilanda et al. (2019)
		b) Inhibit protease enzyme of HIV-I	
Mangiferin	*Mangifera indica* L.	Inhibit HSV-1 virus duplication	Al-Rawi et al. (2019)
(+)-pinoresinol 4-O-(6″-O-vanilloyl)-β-d-glucopyranoside	*Calotropis gigantea* (L.) Dryand.	Inhibit NF-κB pathway and viral ribonucleoproteins nuclear export of H1N1 virus	Parhira et al. (2014)
Quercetin, catechin hydrate, and kaempferol	*Aloe vera* (L.) Burm.f.	a) Inhibit influenza-A virus (H1N1 or H3N2), induce autophagy and inhibit M2 viral mRNA synthesis, and M2 protein expression	Choi et al. (2019); Goyal et al. (2020); Khaerunnisa et al. (2020); Solnier and Fladerer (2020)
		b) Inhibit Mpro of SARS CoV-2	
		c) Quercetin inhibited ACE2 receptor of SARS CoV-2	
Feralolide	*Aloe vera* (L.) Burm.f.	Inhibit the main protease (3CLpro) responsible for the replication of SARS-CoV-2	Mpiana et al. (2020)
Coumestan	*Eclipta prostrata L.*	Inhibit HCV NS5B protein leading to RNA replication	Kaushik-Basu et al. (2008)
kaempferol-3-*O*-(6″-*O*-*E*-*p*-coumaroyl)-β-d-glucopyranoside	*Bombax ceiba L.*	a) Inhibit cytopathic effect of RSV	Schwarz et al. (2014); Zhang et al. (2015); Ren et al. (2020)
		b) Inhibit ORF 3a protein of SARS-CoV-2 leading to interference of virus release mechanism and reduce apoptosis	
Anolignan A Anolignan B	*Anogeissus acuminata (Roxb. ex DC.) Wall. ex Guillem. & Perr*	Inhibit HIV-I reverse transcriptase (RT)	El-Ansari et al. (2020)
Humulene epoxide	*Cyperus rotundus L.*	Inhibit four target proteins of SARS-CoV-2 such as spike glycoprotein, papain-like protease (PLpro), 3-chymotrypsin-like protease (3CLpro) and RNA-dependent RNA polymerase (RdRp)	Amparo et al. (2021)
(+)-catechin	*Albizia procera* (Roxb.) Benth.	a) Inhibit integrase enzyme of human influenza virus-I by interacting with Thr66, Gly148, and Glu152.	Panthong et al. (2015); Khaerunnisa et al. (2020)
		b) Inhibit Mpro enzyme of SARS CoV-2	
Flacourtosides A and E Betulinic acid 3β-caffeate	*Flacourtia indica* (Burm.f.) Merr.	Inhibit DENV RNA polymerase	Bourjot et al. (2012)
(+)-cycloolivil-4′-O-β-d-glucopyranoside	*Swertia angustifolia* var. pulchella (D. Don) Burkill	Inhibit HBsAg and HBeAg secretion and HBV DNA replication	Zhou et al. (2015)
Ursolic acid	*Ocimum tenuiflorum* L. *Ocimum basilicum* L. *Ocimum gratissimum* L.	a) Inhibit replication of HSV-I and II	Tshilanda et al. (2020)
		b) Inhibit multiplication of HCV	
Apigenin	*Ocimum basilicum* L.	a) Inhibit ACE2 receptor and 3CLpro of SARS-CoV-2	Benencia and Courreges (2000); Goyal et al. (2020)
		b) Inhibit replication of HBV	
Rosmarinic acid	*Ocimum tenuiflorum* L. *Ocimum americanum* L.	a) Inhibit replication of HSV-I and II	Tshilanda et al. (2020)
		b) Inhibit protease enzyme of HSV-I and II	
β-caryophyllene	*Ocimum campechianum* Mill.	Inhibit replication of HSV-I and II	Tshilanda et al. (2020)
Trans-anethole	*Ocimum carnosum (Spreng.) Link & Otto ex Benth.*	Inhibit multiplication of HSV-I and II	Astani et al. (2010)
Ajoene	*Allium sativum* L.	a) Prevent HIV-induced destruction of CD + cells	Rouf et al. (2020)
		b) Enhance cellular immunity	
		c) Inhibit virus-cell attachment and viral reverse transcriptase of HIV-I	
		d) Induce apoptosis of HCMV infected cells	
Allicin	*Allium sativum* L.	a) Inhibit the entry of HSV-I and II, PIV-3, VV, VSV and HRV-2 by disrupting viral envelope and cell membrane	Rouf et al. (2020)
		b) Inhibit the replication of REV by downregulation of ERK/MAPK pathway	
Alliin, diallyl sulfide, and garlicin	*Allium sativum* L.	Inhibit DENV by diminishing inflammation by suppressing oxidative stress	Rouf et al. (2020)
Allitridin	*Allium sativum* L.	a) Inhibit viral DNA synthesis through inhibition of immediate-early antigen expression of HCMV	Rouf et al. (2020)
		b) Inhibit viral replication by suppressing viral IEG gene transcription	
		c) Enhance Treg expansion and Treg-mediated anti-HCMV immunosuppression	
Gedunin	*Azadirachta indica* A. Juss.	Inhibit NS3 RNA polymerase and NS3 protease helicase (mediate the synthesis of DENV proteins and genetic materials in the host cell) as well as capsid and envelope proteins (required for entry of DENV into host cells)	Rao and Yeturu, (2020)
Phyllanthin and hypophyllantin	*Phyllanthus niruri L.*	Bind to 4GAG protein of HCV leading to interference in viral entry to host cells	Wahyuni et al. (2019)
Piperine	*Piper longum* L.	Inhibit the secretion of HBsAg and HBeAg of HBV	Jiang et al. (2013)
Guaiol	*Piper nigrum* L.	Inhibit 6LU7 and 7JTL of SARS-CoV-2	Pandey et al. (2021)
Seselin	*Aegle marmelos* (L.) Corrêa	Inhibit the receptors SARS-CoV-2S protein, COVID-19 main protease, and free enzyme of the SARS-CoV-2 (2019-nCoV) main protease	Nivetha et al. (2021)
Hesperidin	*Citrus sinensis* (L.) Osbeck	Inhibit ACE2 receptor, RdRp, spike protein and Mpro of SARS-CoV-2 (under clinical trials, phase-II)	Bellavite and Donzelli (2020); Goyal et al. (2020)
Epigallocatechin-3-gallate (EGCG)	*Camellia sinensis* (L.) Kuntze	a) Inhibit Mpro enzyme, and S protein-receptor interaction of SARS CoV-2	Xu et al. (2017); Goyal et al. (2020); Khaerunnisa et al. (2020); Henss et al. (2021)
		b) Inhibit HIV reverse transcriptase by downregulation of the expression of the HIV p24 antigen	
		c) inhibit RNA and DNA synthesis and antigen expression in HBV	
		d) Block the attachment of HIV-I and HSV-I to of host cells	
Epicatechin gallate (ECG)	*Camellia sinensis* (L.) Kuntze	Inhibit Mpro enzyme of SARS CoV-2	Goyal et al. (2020); Khaerunnisa et al. (2020)
6-Gingerol	*Zingiber officinale* Roscoe	Inhibit SARS CoV-2 by interacting viral proteases, RNA binding protein and Spike protein	Rathinavel et al. (2020)
Gingeronone A	*Zingiber officinale* Roscoe	Inhibit 6LU7 and 7JTL of SARS-CoV-2	Pandey et al. (2021)
Curcumin	*Curcuma Longa* L.	a) SARS-CoV-2: Inhibit ACE2 receptor, viral replication and Mpro	Jennings and Parks (2020); Khaerunnisa et al. (2020); Manoharan et al. (2020)
		b) HIV: Inhibit replication and degrade viral protein	
		c) DENV: Inhibit viral entry, replication and protease enzyme	
		d) IAV: Inhibit replication	
		e) EV 71: Downregulation of protein expression	
		f) ZIKV, CHIKV, VSV, and RSV: Inhibit viral attachment to host cell surface	

## Mechanistic Insight of Antiviral Activities of the Plant Metabolites Derived From Medicinal Plants

From the ancient times, medicinal plants are considered as one of the major priorities of treating illness. Search of antiviral drugs from plant sources is crucial due to fatality and repeated mutations of viruses. Apart from these, new and deadly viral strains are infecting humans time to time. In the last few decades, advancement of synthetic medicinal chemistry has shed light on discovery of synthetic antiviral drugs. A number of synthetic antiviral drugs have been developed which are effective against numerous viruses. Unfortunately, these drugs produce serious adverse effects for continuous administrations. Moreover, many of the synthetic antiviral drugs are ineffective against mutant or resistant strains of viruses. Therefore, the demand for non-toxic antiviral drugs having efficiency to cure viral infections completely still persists. Due to scientific evidences on antiviral potential of naturally produced compounds and their mild side-effects, researchers place their attention extensively on natural resources, especially on plants to search for bioactive metabolites with potent antiviral activities and adequate drug-properties. Pharmaceuticals and nutraceuticals are also paying attention to herbal preparations by using crude extract, syrup, essential oil, and gel extracted from medicinal plants. Interestingly, in recent years, these industries have manufactured them as commercial drug products to treat specific diseases (Table 4).

**TABLE 4:** Available commercial herbal preparations from antiviral medicinal plants in Bangladesh

Product	Species	Used part	Name of the Company
Kalomegh	*Andrographis paniculata* (Burm.f.) Nees	Leaf	Square Herbal and Nutraceuticals Ltd.
			ACME Laboratories Ltd.
Tulsi	*Ocimum tenuiflorum* Burm. f.	Leaf	Square Herbal and Nutraceuticals Ltd.
			ACME Laboratories Ltd.
Bashak	*Justicia adhatoda* L.	Leaf	Square Laboratories Ltd.
			ACME Laboratories Ltd.
Garlic oil	*Allium sativum* L.	Bulb	Square Herbal and Nutraceuticals Ltd.
Chirata	*Swertia angustifolia* var. pulchella (D. Don) Burkill	Whole plant	Drug International Ltd.
Aloe vera gel	*Aloe vera* (L.) Burm.f.	Leaf	Drug International Ltd.

At present, the outbreak of COVID-19 has turned into an evolving worldwide health crisis. Few years back, ZIKV, EV, DENV, and CHIKV have affected a lot of people. Along with these, HIV infection and its treatment still remain unresolved. About 46 medicinal plants available in Bangladesh have been enlisted to have broad-spectrum antiviral activities against a number of viruses. Though phytochemical profiles of these plants are not yet revealed completely, 36 of bioactive metabolites have been reported to exhibit potential antiviral activities with revealing the underlying mechanisms of their activities. Table 3 showed their sources and potential mechanism of activities.

### Effects on SARS-CoV-2

**FIGURE 1:**
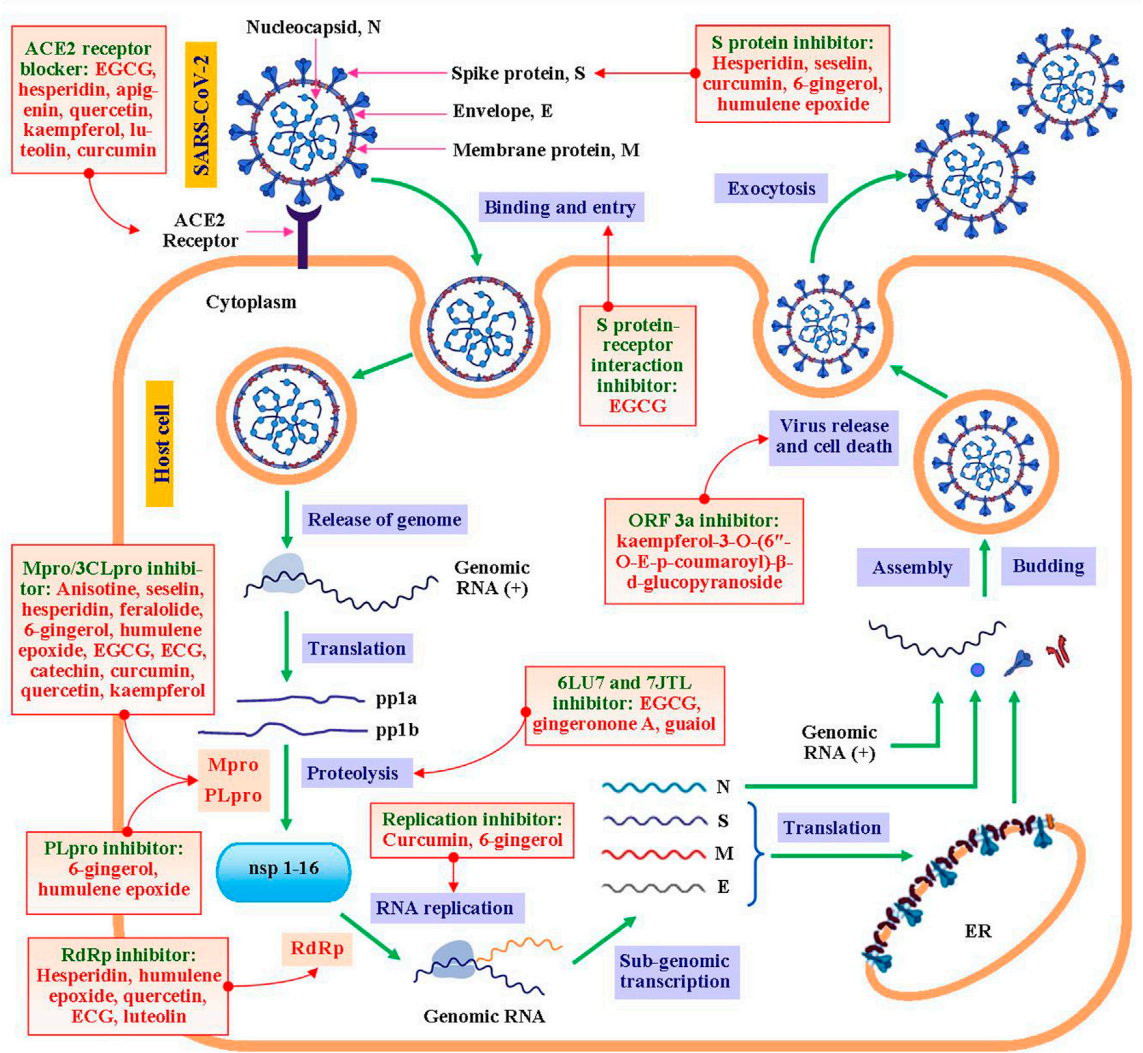
Impact of bioactive metabolites derived from Bangladeshi medicinal plants on molecular targets of various steps of multiplication process of SARS-CoV-2. ACE2, angiotensin converting enzyme 2; N, Nucleocapsid; S, M, E:,spike, membrane, envelope proteins; pp1a, pp1b, nonfunctional polypeptides; nsp, nonstructural proteins; Mpro, main protease; 3CLpro, 3-chymotrypsin like protease; PLpro, papain like protease; RdRp, RNA dependent RNA polymerase; RNA (+), positive-sense RNA; and ER, endoplasmic reticulum.

COVID-19, considered as the deadliest viral infection in present time worldwide. SARS-CoV-2 is the responsible strain belonging to β-coronavirus genus which is spherical shaped enveloped virus packed with single stranded positive-sense (+) genomic RNA. It contains ultra-structural spike proteins on the surface having crown resembled shape (corona) appearance. The genome of this virus encodes structural, accessory, and non-structural proteins. Nucleocapsid (N), spike protein (S), membrane protein (M), and envelope protein (E) are the major structural proteins (Haake et al., 2020). The multiplication of this virus involves several steps mediated by numerous functional molecules which might be important targets for development of the drug therapy for this virus (V’kovski et al., 2021). These cellular and molecular targets of coronavirus can be inhibited and/or interfered by bioactive metabolites derived from medicinal plants found in Bangladesh (Figure 1).

This multiplication process is initiated by viral attachment to host cell surface followed by endocytosis via binding and interaction of viral S protein to angiotensin converting enzyme-2 receptor (ACE2 receptor) on the host cell surface. Inhibitor of S protein, blocker of ACE2 receptor or interferer of S protein-ACE2 receptor interaction might inhibit viral entry to host cell. A number of *in silico* studies demonstrated that numerous metabolites derived from Bangladeshi medicinal plants including (a) hesperidin, seselin, 6-gingerol, and humulene epoxide interacted with and inhibited S protein of SARS-CoV-2 (Bellavite and Donzelli, 2020; Rathinavel et al., 2020; Amparo et al., 2021; Nivetha et al., 2021); (b) hesperidin, kaemferol, apigenin, luteolin, quercetin, and curcumin inhibited ACE2 receptor (Goyal et al., 2020; Manoharan et al., 2020); and (c) hesperidin, seselin, EGCG, curcumin, 6-gingerol, and humulene epoxide interfered with the interaction of S protein-receptor (Henss et al., 2021). These metabolites might be considered for development of potential SARS-CoV-2 entry inhibitors. After endocytosis, the genomic RNA gets translated to nonfunctional polypeptides which are cleaved to functional proteins via proteolytic activity of Mpro/3CLpro and PLpro enzymes. Anisotine, hesperidin, seselin, feralolide, 6-gingerol, humulene epoxide, catechin, ECG, EGCG, curcumin, quercetin, and kaempferol have been reported for exhibiting inhibition potential for main protease, Mpro/3CLpro enzyme (Bellavite and Donzelli, 2020; Khaerunnisa et al., 2020; Mpiana et al., 2020; Rathinavel et al., 2020; Amparo et al., 2021; Ghosh et al., 2021; Nivetha et al., 2021); whereas 6-gingerol and humulene epoxide inhibited PLpro enzyme (Rathinavel et al., 2020; Amparo et al., 2021) resulting inhibition of proteolysis and ending with non-infective nonfunctional proteins. Apart from these, gingeronone A and guaiol inhibited 6LU7 and 7JTL which are crucial for proteolysis mechanism (Pandey et al., 2021). The next step involves replication of genomic RNA from 16 types of nonstructural proteins (nsp 1-16) regulated by RNA dependent RNA polymerase (RdRp) which was inhibited by hesperidin, luteolin, quercetin, ECG, and humulene epoxide (Goyal et al., 2020; Amparo et al., 2021). According to latest researches regarding drug development against SARS-CoV-2 virus, ACE2 receptor blockers as well as RdRp enzyme inhibitors are considered as the most important candidates. Currently, hesperidin is under phase-II clinical trials for treatment of COVID-19 due to its potential activities against these two major targets.

Besides, curcumin and 6-gingerol have been reported for inhibiting this replication process (Khaerunnisa et al., 2020; Rathinavel et al., 2020). After translation and post-translational maturation, the genomic RNA and proteins get assembled, and initiated exocytosis mechanism resulting apoptosis of host cell. Kaempferol-3-O-(6″-O-E-p-coumaroyl)-β-d-glucopyranoside, a plant-derived bioactive compound inhibited ORF 3a, a viral protein of coronavirus involves in release mechanism (SARS-CoV-1) as well as induction of apoptosis (SARS-CoV-2) (Schwarz et al., 2014; Ren et al., 2020).

### Effects on HIV

HIV, considered as one of the most fatal virus which causes acquired immunodeficiency syndrome (AIDS). This virus attacks CD4^+^ lymphocytes which lead to cell death and resultant immune deficiency. Thus, invention of antiretroviral therapy to combat this virus remains one of the global challenges to researchers. Multiplication of this virus involves several basic steps, such as attachment to host cell surface, entry and uncoating of genetic materials to the host cell, reverse transcription of genomic RNA with the help of reverse transcriptase (RT) enzyme followed by translocation of the DNA to host nucleus. Then, the viral DNA gets integrated into host genome and undergoes transcription resulting formation of mRNA and genetic RNA. The mRNA undergoes translation to form viral proteins which are assembled accompanied by genetic RNA in form of virion. These newly formed virions are released from host cells by rapturing plasma membranes and got matured by the help of protease enzyme (Kirchhoff, 2013).

**FIGURE 2:**
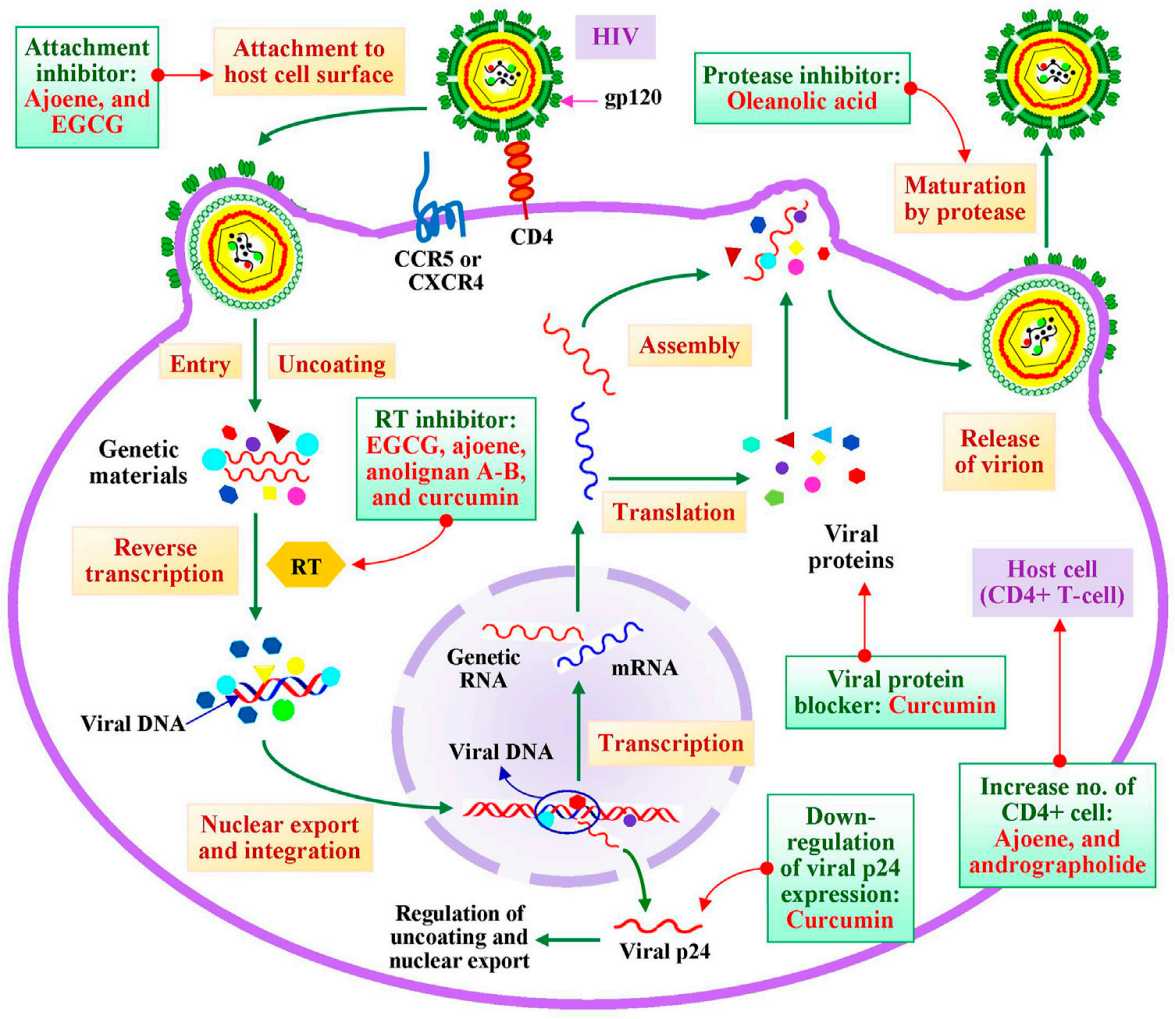
Impact of bioactive metabolites derived from Bangladeshi medicinal plants on molecular targets of various steps of multiplication process of HIV. RT, reverse transcriptase; EGCG, epigallocatechin-3-gallate; and gp120, glycoprotein-120.

Numerous bioactive metabolites have been tested and reported for having efficacy to block the steps of multiplications of this virus (Figure 2). Researchers demonstrated that interaction of gp120 of HIV and CD4 receptor of host cell surface has been inhibited by EGCG and ajoene (Williamson et al., 2006; Rouf et al., 2020). Reverse transcription is one of the major molecular targets of discovery of antiviral drugs against HIV. Bangladeshi medicinal plant-derived biomolecules anolignan-A, anolignan-B, ajoene, and EGCG inhibited this step by inhibiting RT enzyme. EGCG inhibits this step by interfering Nrf2, AKT, and AMPK signaling transduction pathways which are essential for regulation of viral replication. (Li et al., 2011; Zhang et al., 2012; El-Ansari et al., 2020; Rouf et al., 2020). Besides, this biomolecule affects uncoating and nuclear translocation of genetic materials indirectly by downregulation of the expression of p24 gene (Xu et al., 2017). Synthesized viral proteins are essential components for formation of new virions. Curcumin, found in *Curcuma longa*, has been reported for degradation of newly synthesized viral proteins (Jennings and Parks, 2020). Maturation of newly released virions is mandatory for attaining infectivity which involves protease enzyme-regulated proteolytic cleavage. This protease enzyme is inhibited by oleanolic acid (Tshilanda et al., 2019). Apart from these, immune deficiency is observed in HIV-infected patients because of decreasing the number of CD4^+^ lymphocytes which is actually the results of plasma membrane disruption and subsequent cell death. Scientific research showed that ajoene blocked HIV-induced CD4^+^ cell destruction (Rouf et al., 2020). Another study stated that adrographolide treatment increased the CD4^+^ cell counts in HIV-positive patients investigating under phase-I clinical trial (Calabrese et al., 2000).

### Effects on HBV

**FIGURE 3:**
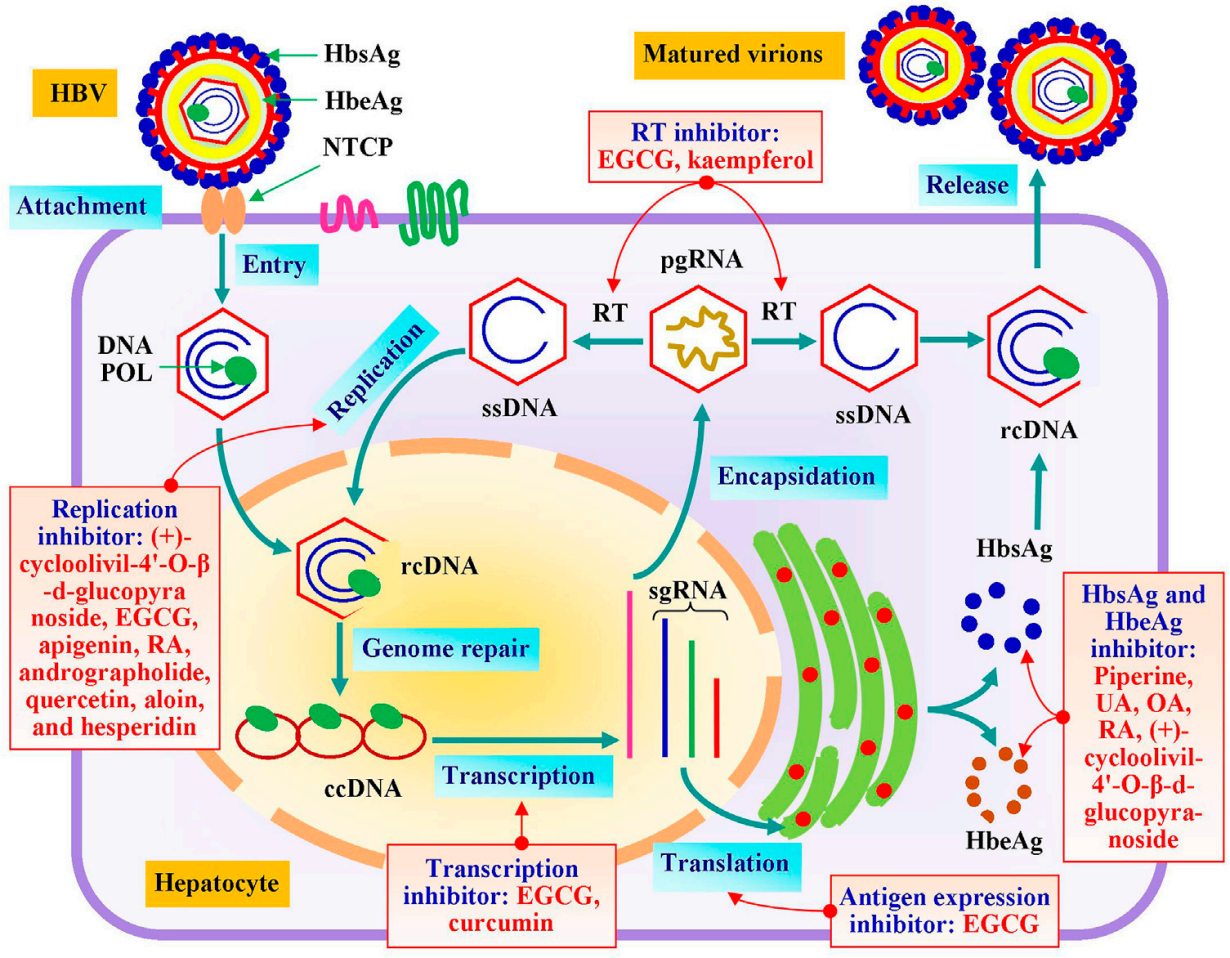
Impact of bioactive metabolites derived from Bangladeshi medicinal plants on molecular targets of HBV. NTCP, Na^+^-taurocholate co-transporting polypeptide; DNA POL, DNA polymerase; RT, reverse transcriptase; rcDNA, relaxed circular DNA; ccDNA, closed circular DNA; pgRNA, pre-genomic RNA; HbsAg, hepatitis-B surface antigen; HbeAg, hepatitis-B e antigen; RA, rosmarinic acid; UA, ursolic acid; and OA, oleanolic acid.

HBV is a unique type of virus that attacks the hepatocytes resulting severe liver infection. Its genomic material is partially double-stranded DNA, commonly known as relaxed-circular DNA or rcDNA. The multiplication process of this virus is distinctive which involves complex and sequential stages (Grimm et al., 2011). It initiates with viral attachment to Na+-taurocholate co-transporting polypeptide (NTCP), also known as sodium/bile acid cotransporter present on plasma membrane leading to genomic entry to hepatocytes. The genomic rcDNA gets translocated to nucleus where host proteins and enzymes repair it by covalent ligation of DNA double strands and form complementary closed circular DNA or ccDNA. It is a highly stable molecular template that exhibits capability to modulate the progression status of severe and barely curable chronic liver infection. After that, the transcription of ccDNA generates subgenomic RNA (sgRNA) and pre-genomic RNA (pgRNA). Bioactive molecules, such as EGCG and curcumin have been reported to inhibit this transcription step leading to reduction of viral load (Xu et al., 2017; Jennings and Parks, 2020) (Figure 3).

This multiplication cycle proceeds by translation and processing of viral antigen particles from sgRNA. Hepatitis-B surface antigen (HbsAg) and hepatitis-B e antigen (HbeAg) are predominantly used screening parameter to assay anti-HBV activity. HbsAg is essential for viral assembly whereas HbeAg is a circulating protein in blood of infected patients during active replication stage. This HbeAg level is an indicator of predicting that the patient is whether infectious to others or not (Grimm et al., 2011). Piperine, ursolic acid, oleanolic acid, and (+)-cycloolivil-4′-O-β-d-glucopyranoside have been testified to decrease the synthesis of HbsAg and HbeAg in many studies (Zhou et al., 2015; Tshilanda et al., 2019; Liu et al., 2020). Quercetin has also been reported to decrease their synthesis by 60% (Parvez et al., 2020). Besides, EGCG diminished the expression of these antigens significantly (Xu et al., 2017). Alternatively, the pgRNA undergoes encapsidation followed by a complex process of reverse transcription to form single strand (-) DNA or ssDNA. This step was inhibited by EGCG and kaempferol in various investigations (Xu et al., 2017; Choi et al., 2019). This ssDNA goes through replication process to generate rcDNA which gets recycled and/or gets assembled along with viral proteins to form new virions. A number of plant-derived metabolites namely (+)-cycloolivil-4′-O-β-d-glucopyranoside, EGCG, aloin, quercetin, apigenin, rosmarinic acid, andrographolide, and hesperidin have been substantiated to interfere the replication process (Lin et al., 2008; Cheng et al., 2015; Zhou et al., 2015; Xu et al., 2017; Wang et al., 2018; Parvez et al., 2019a; Parvez et al., 2019b).

## Challenges and Limitations

Plant metabolites possess multiple therapeutic activities. They can produce synergistic effects resulting superior therapeutic outcomes. Along with numerous advantages, a number of challenges must be overcome during drug discovery process. The major hindrance is drugability of the plant metabolites. Pharmacokinetic ADME (absorption, distribution, metabolism and elimination) parameters are the crucial factors that affect the drugability of a plant-derived compound. Fortunately, advancement of novel drug delivery systems and nanotechnologies enlighten the hope of developing plant metabolites as potential drugs. Already a number of plant metabolites have been formulated as novel drug delivery systems (Table 5). Other impending challenges are (a) procurement and authentication of plant materials; (b) application of high-throughput screening bioassays and scale-up of bioactive lead compounds; and (c) complexity in isolation and purification processes (Jachak and Saklani, 2007). Moreover, the toxicities of plant metabolites are sometimes overlooked during laboratory based assays which are observed during clinical trials. Isolation, purification, and bioassay of pure plant-derived compounds are relatively complex, time consuming and required so much efforts, thus failure of drug development at clinical trial phases are very disappointing (Phu et al., 2020).

**TABLE 5:** Effective delivery systems for oral delivery of plant metabolites with antiviral activity (Ben-Shabat et al., 2020).

Plant metabolite	Novel delivery system
Andrographolide	Self-microemulsion, microsphere, nanosuspension, self-nanodispersion, nanoparticle, and inclusion complex
Oleanolic acid	Self-microemulsion, nanoparticle, self-nanoemulsion and nanosuspension
Quercetin	Nanocrystal, nanoparticle, phytosome, nanoliposome, self-nanoemulsion, mixed micelle, nanoemulsion, and nanosuspension
Apigenin	W/O/W emulsion, O/W microemulsion, solid dispersion, mixed micelle, micropellet, phytosome, and self-microemulsion
Curcumin	Mixed micelle, nanoparticle, solid dispersion, self-nanoemulsion, self-microemulsion, lipid carrier, co-polymeric micelle, and exosome

W/O/W, water-in-oil-in-water, O/W, oil-in-water.

## Concluding Remarks

In this review, we have summarized the overview of 46 antiviral medicinal plants from 25 families cultivated and originated in Bangladesh. In most of the cases, medicinal plants are screened by preliminary *in vitro and/or in silico* assays for antiviral activities, but very few of them are moved forward for further studies and clinical trials. Moreover, bioactive phytochemicals are not profiled for all of these plants. From the available data regarding these plants, a total of 79 compounds with antiviral activities have been found. Amongst them, about 37 bioactive compounds have significant antiviral activities accompanied by mechanistic explanation. These compounds showed potential inhibitory activities against SARS-CoV-2, HIV, HBV, HCV, HSV, DENV, influenza virus and so others. EGCG, oleanolic acid, hesperidin, quercetin, curcumin, kaempferol, and andrographolide showed activity against multiple viruses. Adequate studies are not available regarding structure activity relationship of these bioactive compounds which are crucial to develop drugs active against fatal viruses. Thus, for the development of desired antiviral drug molecules from these medicinal plants, further investigations should be necessary to unveil the mechanism of antiviral activities of the isolated bioactive metabolites along with enlightenment of the structure activity relationship.
